# Supplementary material for: Hospital-Based Influenza and Pneumococcal Vaccination for Cancer Patients on Active Treatment and Their Family Members during the COVID-19 Pandemic in Italy: A Single-Center Experience
Source: Vaccines (Basel). 2024 Jun 8;12(6):642. doi: 10.3390/vaccines12060642 (PMC11209258; doi:10.3390/vaccines12060642)
Supplement: Supplementary file 1 [file vaccines-12-00642-s001.zip › vaccines-3009336-supplementary.pdf]

**Table S1.** Outcomes of patients according to treatment exposure

| | **Naive (n=89)** | **Pretreated (n=105)** | **OR* (95% CI) Naive vs pretreated** | **p-value** |
|---|---|---|---|---|
| **Age, median (IQR)** | 63 (53, 74) | 63 (50, 72) | | |
| **Age > 65 yo** | 42 (47.2%) | 45 (42.9%) | | |
| **Gender** | | | | |
| **Male** | 35 (39.3%) | 28 (26.7%) | | |
| **Female** | 54 (60.7%) | 77 (73.3%) | | |
| **ILI** | 7 (7.9%) | 11 (10.5%) | 0.74 (0.27, 2.02) | 0.56 |
| **ED/HA** | 2 (2.3%) | 1 (1.0%) | 1.55 (0.13, 18.9) | 0.73 |
| **Local AE to I vaccine** | 27 (30.3%) | 39 (37.1%) | 0.77 (0.42, 1.41) | 0.40 |
| **Local AE to P vaccine** | 24 (28.2%) | 42 (41.6%) | 0.61 (0.32, 1.15) | 0.12 |
| *missing* | *4 (4.5%)* | *4 (3.8%)* | | |
| **Overall systemic AE** | 13 (14.6%) | 24 (22.9%) | 0.64 (0.29, 1.38) | 0.25 |

*Logistic regression analysis adjusted for age and gender

Abbreviations: *ILI*, influenza-like illness; *ED*, access to the emergency department; *HA*, hospital admission; *I*, influenza; *AE*, adverse events, *P*, pneumococcal infection.

**Table S2.** Outcomes of patients according to type of treatment

| | **Hormone-therapy (n=27)** | **Chemotherapy and target therapy (n=34)** | **Target therapy (n=59)** | **Chemotherapy (n=74)** | **p-value[A]** | **p-value*** |
|---|---|---|---|---|---|---|
| **Age, median (IQR)** | 71 (62, 76) | 64 (46, 70) | 60 (50, 70) | 66 (53, 73) | 0.02 | |
| **Age > 65 yo** | 16 (59.3%) | 15 (44.1%) | 18 (30.5%) | 38 (51.4%) | | |
| ***Gender*** | | | | | | |
| **Males** | 10 (37.0%) | 14 (41.2%) | 14 (23.7%) | 25 (33.8%) | | |
| **Females** | 17 (63.0%) | 20 (58.8%) | 45 (76.3%) | 49 (66.2%) | | |
| **ILI** | 3 (11.1%) | 4 (11.8%) | 5 (8.5%) | 6 (8.1%) | 0.87 | 0.92 |
| **ED/HA** | 1 (3.7%) | 1 (2.9%) | 1 (1.7%) | 0 (0.0%) | 0.24 | 0.35 |
| **Local AE to I vaccine** | 8 (29.6%) | 8 (23.5%) | 22 (37.3%) | 28 (37.8%) | 0.44 | 0.43 |
| **Local AE to P vaccine** | 5 (21.7%) | 15 (44.1%) | 22 (38.6%) | 24 (33.3%) | 0.34 | 0.32 |
| *missing* | *4 (14.8%)* | *0 (0.0%)* | *2 (3.4%)* | *2 (2.7%)* | | |
| **Overall systemic AE** | 3 (11.1%) | 8 (23.5%) | 15 (25.4%) | 11 (14.9%) | 0.27 | 0.26 |

[A]Kruskal-Wallis, chi-square o Fisher's exact test

*Logistic regression analysis adjusted for age and gender

Abbreviations: *ILI*, influenza-like illness; *ED*, access to the emergency department; *HA*, hospital admission; *I*, influenza; *AE*, adverse events, *P*, pneumococcal infection.

**Table S3.** Outcomes of patients according to schedule of treatment

| | Weekly (n=22) | Bi-weekly (n=15) | Tri-weekly (n=106) | continuous administration (n=51) | p-value[A] | p-value* |
|---|---|---|---|---|---|---|
| **Age, median (IQR)** | 53 (45, 69) | 70 (59, 75) | 63 (52, 74) | 65 (65, 71) | 0.14 | |
| **Age > 65 yo** | 7 (31.8%) | 10 (66.7%) | 47 (44.3%) | 23 (45.1%) | | |
| ***Gender*** | | | | | | |
| **Male** | 3 (13.6%) | 8 (53.3%) | 36 (34.0%) | 16 (31.4%) | | |
| **Female** | 19 (86.4%) | 7 (46.7%) | 70 (66.0%) | 35 (68.6%) | | |
| **ILI** | 2 (9.1%) | 0 (0.0%) | 9 (8.5%) | 7 (13.7%) | 0.52 | 0.25 |
| **ED/HA** | 0 (0.0%) | 0 (0.0%) | 1 (0.9%) | 2 (3.9%) | 0.60 | 0.43 |
| **Local AE to I vaccine** | 6 (27.3%) | 8 (53.3%) | 33 (31.1%) | 19 (37.3%) | 0.31 | 0.33 |
| **Local AE to P vaccine** | 9 (40.9%) | 3 (21.4%) | 33 (32.4%) | 21 (43.8%) | 0.35 | 0.33 |
| *missing* | *0 (0.0%)* | *1 (6.7%)* | *4 (3.8%)* | *3 (5.9%)* | | |
| **Overall systemic AE** | 5 (22.7%) | 2 (13.3%) | 19 (17.9%) | 11 (21.6%) | 0.85 | 0.84 |

[A]Kruskal-Wallis, chi-square o Fisher's exact test

*Logistic regression analysis adjusted for age and gender

Abbreviations: *ILI*, influenza-like illness; *ED*, access to the emergency department; *HA*, hospital admission; *I*, influenza; *AE*, adverse events, *P*, pneumococcal infection.
